# Identification of hub genes with prognostic values in gastric cancer by bioinformatics analysis

**DOI:** 10.1186/s12957-018-1409-3

**Published:** 2018-06-19

**Authors:** Ting Li, Xujie Gao, Lei Han, Jinpu Yu, Hui Li

**Affiliations:** 1 Department of Gastrointestinal Cancer Biology, Tianjin Medical University Cancer Institute and Hospital, Tianjin, China; 2 Cancer Molecular Diagnostics Core, Tianjin Medical University Cancer Institute and Hospital, Tianjin, China; 3 Key Laboratory of Cancer Immunology and Biotherapy, Tianjin, China; 4 National Clinical Research Center for Cancer, Tianjin, China

**Keywords:** Gastric cancer, Bioinformatics analysis, Differentially expressed genes, Prognosis

## Abstract

**Background:**

Gastric cancer (GC) is a prevalent malignant cancer of digestive system. To identify key genes in GC, mRNA microarray GSE27342, GSE29272, and GSE33335 were downloaded from GEO database.

**Methods:**

Differentially expressed genes (DEGs) were obtained using GEO2R. DAVID database was used to analyze function and pathways enrichment of DEGs. Protein-protein interaction (PPI) network was established by STRING and visualized by Cytoscape software. Then, the influence of hub genes on overall survival (OS) was performed by the Kaplan-Meier plotter online tool. Module analysis of the PPI network was performed using MCODE. Additionally, potential stem loop miRNAs of hub genes were predicted by miRecords and screened by TCGA dataset. Transcription factors (TFs) of hub genes were detected by NetworkAnalyst.

**Results:**

In total, 67 DEGs were identified; upregulated DEGs were mainly enriched in biological process (BP) related to angiogenesis and extracellular matrix organization and the downregulated DEGs were mainly enriched in BP related to ion transport and response to bacterium. KEGG pathways analysis showed that the upregulated DEGs were enriched in ECM-receptor interaction and the downregulated DEGs were enriched in gastric acid secretion. A PPI network of DEGs was constructed, consisting of 43 nodes and 87 edges. Twelve genes were considered as hub genes owing to high degrees in the network. Hsa-miR-29c, hsa-miR-30c, hsa-miR-335, hsa-miR-33b, and hsa-miR-101 might play a crucial role in hub genes regulation. In addition, the transcription factors-hub genes pairs were displayed with 182 edges and 102 nodes. The high expression of 7 out of 12 hub genes was associated with worse OS, including COL4A1, VCAN, THBS2, TIMP1, COL1A2, SERPINH1, and COL6A3.

**Conclusions:**

The miRNA and TFs regulation network of hub genes in GC may promote understanding of the molecular mechanisms underlying the development of gastric cancer and provide potential targets for GC diagnosis and treatment.

**Electronic supplementary material:**

The online version of this article (10.1186/s12957-018-1409-3) contains supplementary material, which is available to authorized users.

## Background

Gastric cancer is the fourth common cancer worldwide, with an estimated 951,600 new cases of gastric cancer (GC) and estimated 723,100 deaths from it in 2012 [1]. In China, GC is one of the most common malignancies and is the third leading cause of cancer death in 2010 [2]. Despite recent improvements in multimodal therapy including surgery, chemotherapy, radiotherapy, and targeted therapy, its overall 5-year survival rate remains below 20% [3]. The abnormal intracellular signaling molecules contribute a part of poor prognosis due to tumor invasion and metastasis [4]. Such signaling molecules, like matrix metalloproteinase (MMP)-2, MMP-9, and vascular endothelial growth factor (VEGF), have been shown to promote tumor metastasis [5]. Moreover, the abnormal expression of non-coding RNA is also an important factor according to recent studies, especially microRNAs (miRNAs) [6]. MiRNAs are a class of evolutionary conserved small RNA that regulates gene expression by targeting mRNAs to translation repression or triggering mRNA degradation. Various biological processes were involved in the regulation network between miRNAs and target mRNAs, including differentiation, proliferation, survival, stress response, and oncogenesis [7–10]. Like miRNAs, transcription factors (TFs) regulate diverse cellular pathways and are widely believed to regulate most biological processes, including cancer. TFs are sequence-specific DNA-binding proteins which act as transcriptional activators and repressors.

In recent years, the application of high-throughput platforms in gene expression (GE) is becoming more valuable in clinical research, like molecular classification, prognosis prediction, and new targeted drug discovery [11–13]. Hundreds of DEGs were shown in many gene expression profiling studies on GC carcinogenesis, which involved in different pathways, biological process, molecular function. However, the interaction network of DEGs remains to be clarified. In this work, three mRNA microarray datasets were analyzed to obtain DEGs between GC tissues and normal tissues. We further explored GC development by a way of DEGs functional enrichment and interaction network analysis, combined with survival analysis and mRNA-miRNA interaction analysis, also constructed gene–transcription factor interaction network to identify key genes in GC.

## Methods

### Identification of DEGs

Three gene expression profiles (GSE27342, GSE29272, and GSE33335) were acquired from GEO database. The array data of GSE27342 consisted of 80 paired GC tissues and adjacent tissues [14]. GSE33335 contained 25 paired GC tissues and adjacent tissues [15]. GSE29272 included 134 paired GC tissues, and adjacent tissues were submitted by Wang et al. [16]. DEG was obtained from GEO database by a way of GEO2R analysis (http://www.ncbi.nlm.nih.gov/geo/geo2r/). The adj. *P* < 0.05 and |logFC| > 1.5 were set as DEGs cutoff criterion.

### Gene ontology and pathway enrichment analysis of DEGs

The Database for Annotation, Visualization and Integrated Discovery (DAVID, http://david.abcc.ncifcrf.gov/) has facilitated the transition from data collection to biological analysis [17]. Gene ontology (GO) and Kyoto Encyclopedia of Genes and Genomes (KEGG) pathway enrichment analysis was performed using DAVID online tool. *P* < 0.01 was set as the cutoff criterion.

### Integration of protein-protein interaction network and modules selection

To explore the interaction of DEGs, we submitted the DEGs to the Search Tool for the Retrieval of Interacting Genes (STRING, http://string.embl.de/) database, and only validated interactions with combined score > 0.4 were selected as significant. Then, integration of protein-protein interaction (PPI) networks were visualized using Cytoscape software. The Molecular Complex Detection (MCODE) was applied to screen modules of PPI network with degree cutoff = 2, node score cutoff = 0.2, k-core = 2, and max. depth = 100 [18]. The functional enrichment and pathway enrichment analysis in the module was performed by DAVID.

### Prediction of stem loop miRNAs for hub genes

miRecords, which is an integrated resource of 11 established miRNA target prediction programs [19], was used to identify the stem loop miRNAs of hub genes. The miRNAs predicted by at least four programs were regarded as the stem loop miRNAs of hub genes.

### Negatively correlated stem loop miRNA of hub genes in gastric cancer

The raw counts of miRNA expression data of 380 gastric cancer tissues were obtained from the TCGA dataset (Illumina HiSeq Systems). MiRNA expression data was normalized by the R/Bioconductor package edgeR [20]. The correlation between hub genes and miRNAs was characterized by the |logFC|. MiRNAs with negative correlation (*P* < 0.05, FDR adjusted *P* < 0.05) were considered as negatively correlated miRNAs.

### Prediction of transcription factor for DEGs

TFs of hub genes were explored combined with the human TF information (NetworkAnalyst, http://www.networkanalyst.ca), recorded using ChIP Enrichment Analysis (ChEA), and visualized using the Cytoscape software [21, 22].

### Survival analysis of DEGs

Kaplan-Meier plotter (KM plotter, http://kmplot.com/analysis/) was capable to assess the survival of 10,188 cancer samples, including 5143 breast, 1648 ovarian, 2437 lung, and 1065 gastric cancer patients with a mean follow-up of 69/40/49/33 months [23]. According to the median expression of a particular gene, the patients with GC were split into high and low expression groups. The overall survival (OS) of GC patients was evaluated using a KM plot. The hazard ratio (HR) with 95% confidence intervals and log rank *P* values were shown on the webpage; then multiple hypothesis testing was calculated.

## Results

### Identification of DEGs

**Fig. 1:**
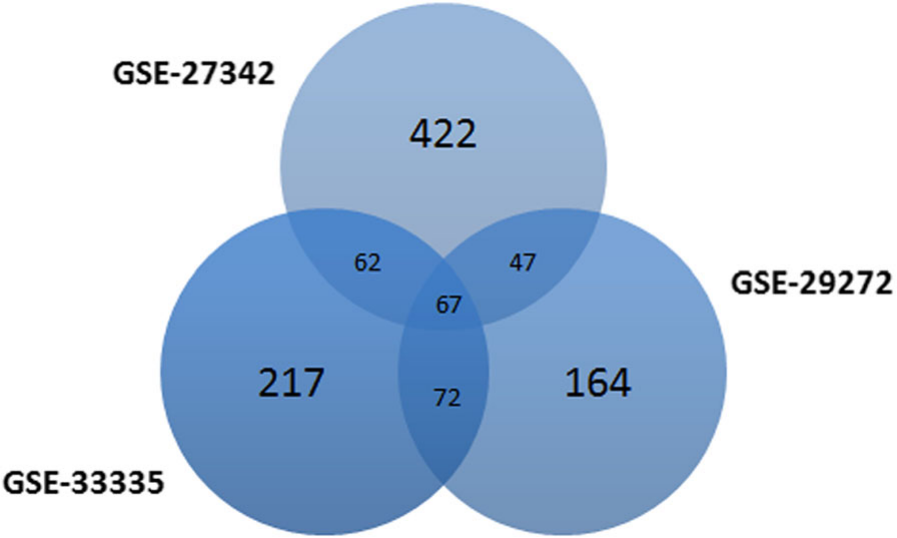
Identification of DEGs in mRNA expression profiling datasets GSE27342, GSE29272, and GSE33335

A total of 598, 350, and 418 DEGs were identified from GSE27342, GSE29272, and GSE33335 datasets. Sixty-seven genes were screened out in all three datasets and were selected for further analysis (Fig. 1). There are 36 upregulated genes and 31 downregulated genes in GC tissues compared to adjacent tissues.

### GO term and KEGG pathway enrichment analysis of DEGs

To gain further insight into the function of identified DEGs, GO term and KEGG pathway enrichment analysis were performed using DAVID. The upregulated genes were mainly enriched in angiogenesis and extracellular matrix organization, while downregulated genes were mainly involved in ion transport and response to bacterium in biological processes (Table 1). Top ten terms of upregulation or downregulation genes were selected and are displayed in Table 1 according to *P* value. Moreover, seven KEGG pathways were overrepresented in upregulated genes, including ECM-receptor interaction, focal adhesion, and PI3K-Akt signaling pathway, and two pathways were identified in downregulation (Table 2).

**Table 1:** GO analysis of DEGs associated with GC

Expression	Term/gene function	Gene count	*P* value
Upregulation	GO:0001568~blood vessel development	9	3.68E-06
	GO:0001944~vasculature development	9	6.21E-06
	GO:0030198~extracellular matrix organization	6	2.43E-05
	GO:0043062~extracellular structure organization	6	2.51E-05
	GO:0030199~collagen fibril organization	4	3.97E-05
	GO:0010033~response to organic substance	13	6.13E-05
	GO:0051291~protein heterooligomerization	4	7.68E-05
	GO:0071230~cellular response to amino acid stimulus	4	8.34E-05
	GO:0072358~cardiovascular system development	9	1.17E-04
	GO:0072359~circulatory system development	9	1.17E-04
Downregulation	GO:0006813~potassium ion transport	4	5.11E-04
	GO:0010107~potassium ion import	3	7.71E-04
	GO:0019731~antibacterial humoral response	3	1.13E-03
	GO:0019730~antimicrobial humoral response	3	1.46E-03
	GO:0042742~defense response to bacterium	4	2.80E-03
	GO:0015672~monovalent inorganic cation transport	4	4.94E-03
	GO:0002803~positive regulation of antibacterial peptide production	2	5.18E-03
	GO:0002760~positive regulation of antimicrobial humoral response	2	5.18E-03
	GO:0002786~regulation of antibacterial peptide production	2	5.18E-03
	GO:0002225~positive regulation of antimicrobial peptide production	2	5.18E-03

**Table 2:** KEGG pathway analysis of DEGs associated with GC

Expression	Term	Count	*P* value
Upregulation	hsa04512: ECM-receptor interaction	7	8.90E-08
	hsa04510: Focal adhesion	7	1.46E-05
	hsa04974: Protein digestion and absorption	5	7.37E-05
	hsa04151: PI3K-Akt signaling pathway	7	2.25E-04
	hsa05146: Amoebiasis	4	3.00E-03
	hsa04670: Leukocyte transendothelial migration	4	4.16E-03
	hsa04514: Cell adhesion molecules (CAMs)	4	5.92E-03
Downregulation	hsa04971: Gastric acid secretion	7	1.64E-08
	hsa04966: Collecting duct acid secretion	3	2.12E-03

### PPI network construction and modules selection

**Fig. 2:**
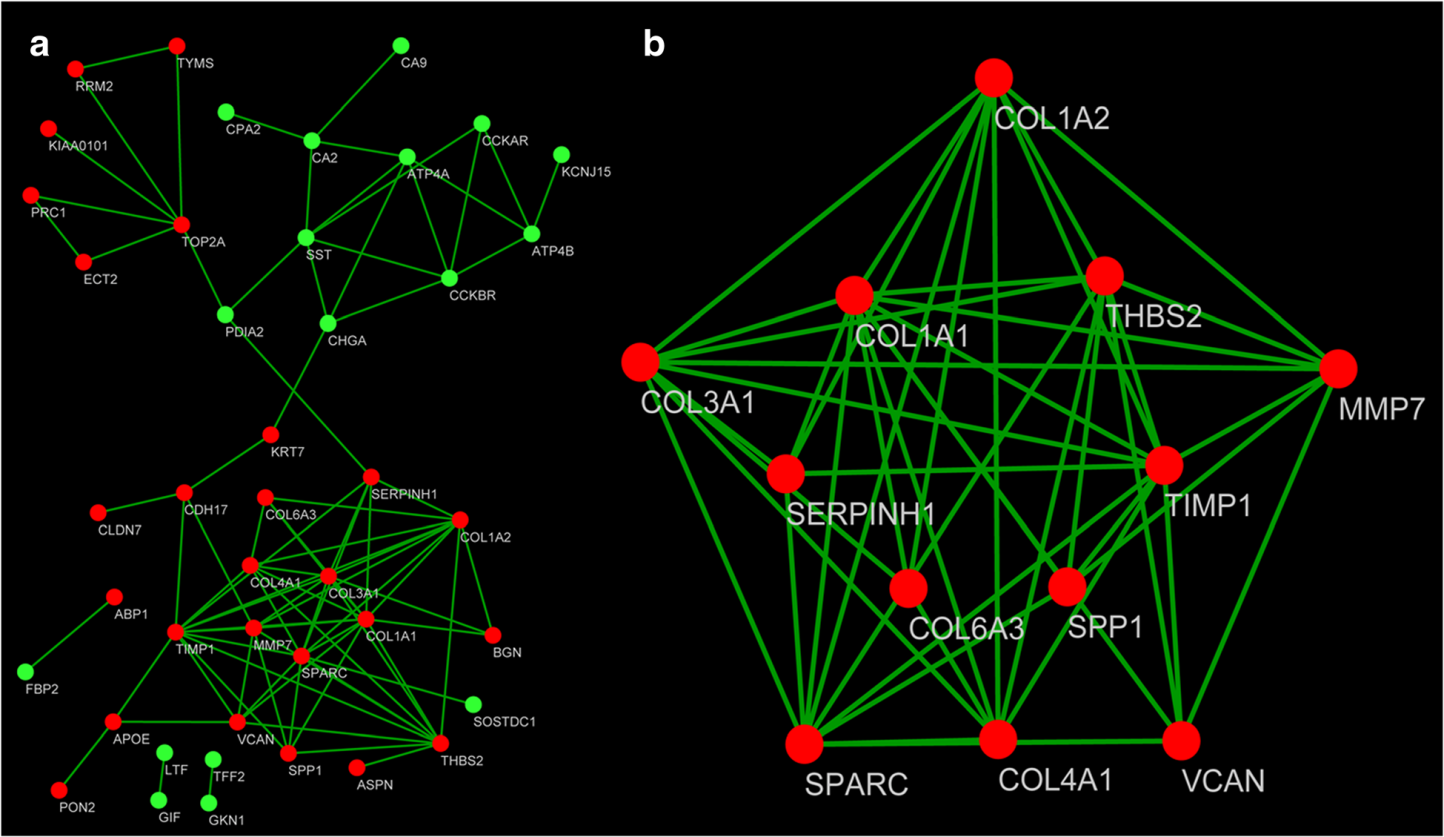
PPI network and a significant module. **a** PPI network of DEGs, red means upregulated genes and green means downregulated genes. **b** A significant module selected from PPI network, all of them were upregulated genes

The PPI network of DEGs is consisted of 43 nodes and 87 edges, including 26 upregulated genes and 17 downregulated genes (Fig. 2a). There are 12 genes selected as hub genes, such as SPP1, TIMP1, MMP7, and COL1A1, enriched in a module when degrees ≥ 8 were set as the cutoff criterion.

A significant module was obtained from PPI network of DEGs using MCODE, including 12 nodes and 45 edges (Fig. 2b). KEGG pathway enrichment analysis revealed that genes in this module were mainly involved in ECM-receptor interaction, focal adhesion, and PI3K-Akt signaling pathway (Table 3).

**Table 3:** KEGG pathways enrichment analysis of the genes in module

Term	Count	*P* value
hsa04512: ECM-receptor interaction	7	3.05E-10
hsa04510: Focal adhesion	7	5.86E-08
hsa04151: PI3K-Akt signaling pathway	7	1.07E-06
hsa04974: Protein digestion and absorption	5	2.63E-06
hsa05146: Amoebiasis	4	2.91E-04
hsa04611: Platelet activation	3	1.15E-02

### MiRNA-hub genes pairs

To investigate the molecular mechanisms underlying the dysregulated hub genes, potential stem loop miRNAs were searched by bioinformatics methods. The stem loop miRNAs of hub genes were predicted by miRecords database. As shown in Table 4, top five predicted stem loop miRNAs of hub genes were displayed according to the prediction program numbers. To validate the accuracy of the predicted stem loop miRNAs for hub genes, we screened the negatively correlated stem loop miRNAs of hub genes in a TCGA dataset composed of 380 gastric cancer tissues (Additional file 1). Top five negatively correlated stem loop miRNAs of hub genes are shown in Table 5 in consideration of logFC. As shown in Table 6, COL1A1, COL1A2, COL4A1, COL6A3, SPARC, and THBS2 might be the targets of hsa-miR-29c. Moreover, COL1A1, COL1A2, COL3A1, COL6A3, SERPINH1, SPARC, and THBS2 were potential targets of hsa-miR-30c. Hsa-miR-335 may regulate COL1A1, COL1A2, COL3A1, COL4A1, COL6A3, SPARC, and THBS2. Hsa-miR-33b possibly bound to 3′ UTR of COL1A1, COL3A1, COL4A1, COL6A3, SPARC, THBS2, and VCAN. But COL1A1, COL3A1, SPARC, SPP1, and THBS2 would be targets of hsa-miR-101 in some ways.

**Table 4:** Hub genes and its predicted miRNAs

Gene	Predicted miRNA	Total
COL1A1	hsa-let-7d hsa-miR-98 hsa-let-7a hsa-let-7 g hsa-let-7i	149
COL1A2	hsa-miR-29c hsa-let-7c hsa-miR-25 hsa-miR-29a hsa-miR-29b	91
COL3A1	hsa-miR-29b hsa-let-7 g hsa-let-7f hsa-let-7i hsa-let-7a	85
COL4A1	hsa-miR-29a hsa-miR-29b hsa-miR-29c hsa-miR-148a hsa-let-7c	118
COL6A3	hsa-miR-29c hsa-miR-29a hsa-miR-29b hsa-miR-206 hsa-miR-330-3p	108
MMP7	hsa-miR-297 hsa-miR-641 hsa-miR-19b hsa-miR-122 hsa-miR-380	32
SERPINH1	hsa-miR-200b hsa-miR-24 hsa-miR-346 hsa-miR-561 hsa-miR-637	56
SPARC	hsa-miR-29a hsa-miR-299-3p hsa-miR-29c hsa-miR-203 hsa-miR-29b	206
SPP1	hsa-miR-220b hsa-miR-127-5p hsa-miR-181c hsa-miR-181a hsa-miR-181b	72
THBS2	hsa-miR-607 hsa-miR-891b hsa-miR-616 hsa-miR-519a hsa-miR-221	184
TIMP1	hsa-miR-484 hsa-miR-892a hsa-miR-1293 hsa-miR-1292	4
VCAN	hsa-miR-203 hsa-miR-144 hsa-miR-632 hsa-miR-135b hsa-miR-136	57

**Table 5:** Hub genes and its screening stem loop miRNAs

Gene	Screening stem loop miRNAs	Total
COL1A1	hsa-miR-378 hsa-miR-144 hsa-miR-29c hsa-miR-7 hsa-miR-556	21
COL1A2	hsa-let-378 hsa-miR-144 hsa-miR-7 hsa-miR-556 hsa-miR-200c	32
COL3A1	hsa-miR-7 hsa-miR-556 hsa-miR-378 hsa-miR-200c hsa-miR-18a	44
COL4A1	hsa-miR-222 hsa-miR-429 hsa-miR-96 hsa-miR-33b hsa-miR-556	23
COL6A3	hsa-miR-200c hsa-miR-96 hsa-miR-7 hsa-miR-592 hsa-miR-200a	38
MMP7	hsa-miR-378 hsa-miR-503 hsa-miR-19a hsa-miR-301b hsa-miR-509	11
SERPINH1	hsa-miR-101 hsa-miR-29c hsa-miR-378 hsa-miR-195	4
SPARC	hsa-miR-7 hsa-miR-378 hsa-miR-18a hsa-miR-19a hsa-miR-96	38
SPP1	hsa-miR-29c hsa-miR-139 hsa-miR-195 hsa-miR-101 hsa-miR-129	6
THBS2	hsa-miR-144 hsa-miR-378 hsa-miR-7 hsa-miR-200c hsa-miR-592	39
VCAN	hsa-miR-378 hsa-miR-556 hsa-miR-200c hsa-miR-7 hsa-miR-96	38

**Table 6:** Hub genes’ negatively correlated stem loop miRNAs in predicted miRNAs

Genes	Negatively correlated stem loop miRNAs	Total
COL1A1	hsa-miR-378 hsa-miR-29c hsa-miR-592 hsa-miR-30c hsa-miR-19a hsa-miR-18a hsa-miR-96 hsa-miR-802 hsa-miR-101 hsa-miR-182 hsa-miR-577 hsa-miR-335 hsa-miR-877 hsa-miR-130b hsa-miR-33b	15
COL1A2	hsa-miR-7 hsa-miR-200c hsa-miR-19a hsa-miR-96 hsa-miR-200a hsa-miR-200b hsa-miR-429 hsa-miR-29c hsa-miR-130b hsa-miR-30c hsa-miR-182 hsa-miR-194 hsa-miR-301b hsa-miR-335 hsa-miR-577 hsa-miR-1304 hsa-miR-105 hsa-miR-196a hsa-miR-579 hsa-miR-9 hsa-miR-135b	21
COL3A1	hsa-miR-200c hsa-miR-96 hsa-miR-144 hsa-miR-200a hsa-miR-200b hsa-miR-429 hsa-miR-130b hsa-miR-877 hsa-miR-192 hsa-miR-182 hsa-miR-33b hsa-miR-301b hsa-miR-577 hsa-miR-335 hsa-miR-30c-2* hsa-miR-1304 hsa-miR-29c hsa-miR-206	22
COL4A1	hsa-miR-429 hsa-miR-96 hsa-miR-33b hsa-miR-556 hsa-miR-200b hsa-miR-200c hsa-miR-182 hsa-miR-18a hsa-miR-29c hsa-miR-19a hsa-miR-301b hsa-miR-192 hsa-miR-335 hsa-miR-378 hsa-miR-130b	15
COL6A3	hsa-miR-509-3p hsa-miR-549 hsa-miR-509-3p hsa-miR-29c hsa-miR-605 hsa-miR-579 hsa-miR-206 hsa-miR-1304 hsa-miR-135b hsa-miR-33b hsa-miR-30c hsa-miR-335 hsa-miR-194 hsa-miR-301b hsa-miR-877 hsa-miR-378 hsa-miR-130b hsa-miR-19a hsa-miR-96	19
MMP7	hsa-miR-19a	1
SERPINH1	hsa-miR-30c hsa-miR-195 hsa-miR-378 hsa-miR-29c	2
SPARC	hsa-miR-579 hsa-miR-605 hsa-miR-135b hsa-miR-215 hsa-miR-767-3p hsa-miR-105 hsa-miR-206 hsa-miR-196a hsa-miR-33b hsa-miR-335 hsa-miR-30c hsa-miR-592 hsa-miR-1304 hsa-miR-101 hsa-miR-182 hsa-miR-222 hsa-miR-188-3p hsa-miR-192 hsa-miR-877 hsa-miR-29c hsa-miR-144 hsa-miR-200a hsa-miR-200c hsa-miR-200b hsa-miR-96 hsa-miR-19a hsa-miR-18a hsa-miR-378	28
SPP1	hsa-miR-195 hsa-miR-101	2
THBS2	hsa-miR-509 hsa-miR-504 hsa-miR-767 hsa-miR-579 hsa-miR-509 hsa-miR-135b hsa-miR-335 hsa-miR-196a hsa-miR-101 hsa-miR-33bhsa-miR-577 hsa-miR-802 hsa-miR-29c hsa-miR-222 hsa-miR-301b hsa-miR-188 hsa-miR-182 hsa-miR-30c hsa-miR-194 hsa-miR-877hsa-miR-192 hsa-miR-130b hsa-miR-206 hsa-miR-429 hsa-miR-96 hsa-miR-19a hsa-miR-200b hsa-miR-18a hsa-miR-486 hsa-miR-556 hsa-miR-592 hsa-miR-200c hsa-miR-144	33
VCAN	hsa-mir-605 hsa-mir-135b hsa-mir-579 hsa-mir-33b hsa-mir-206 hsa-mir-192 hsa-mir-222 hsa-mir-200b hsa-mir-429 hsa-mir-200a hsa-mir-19a hsa-mir-144 hsa-mir-18a hsa-mir-200c	14

### Transcription factors-hub genes pairs

**Fig. 3:**
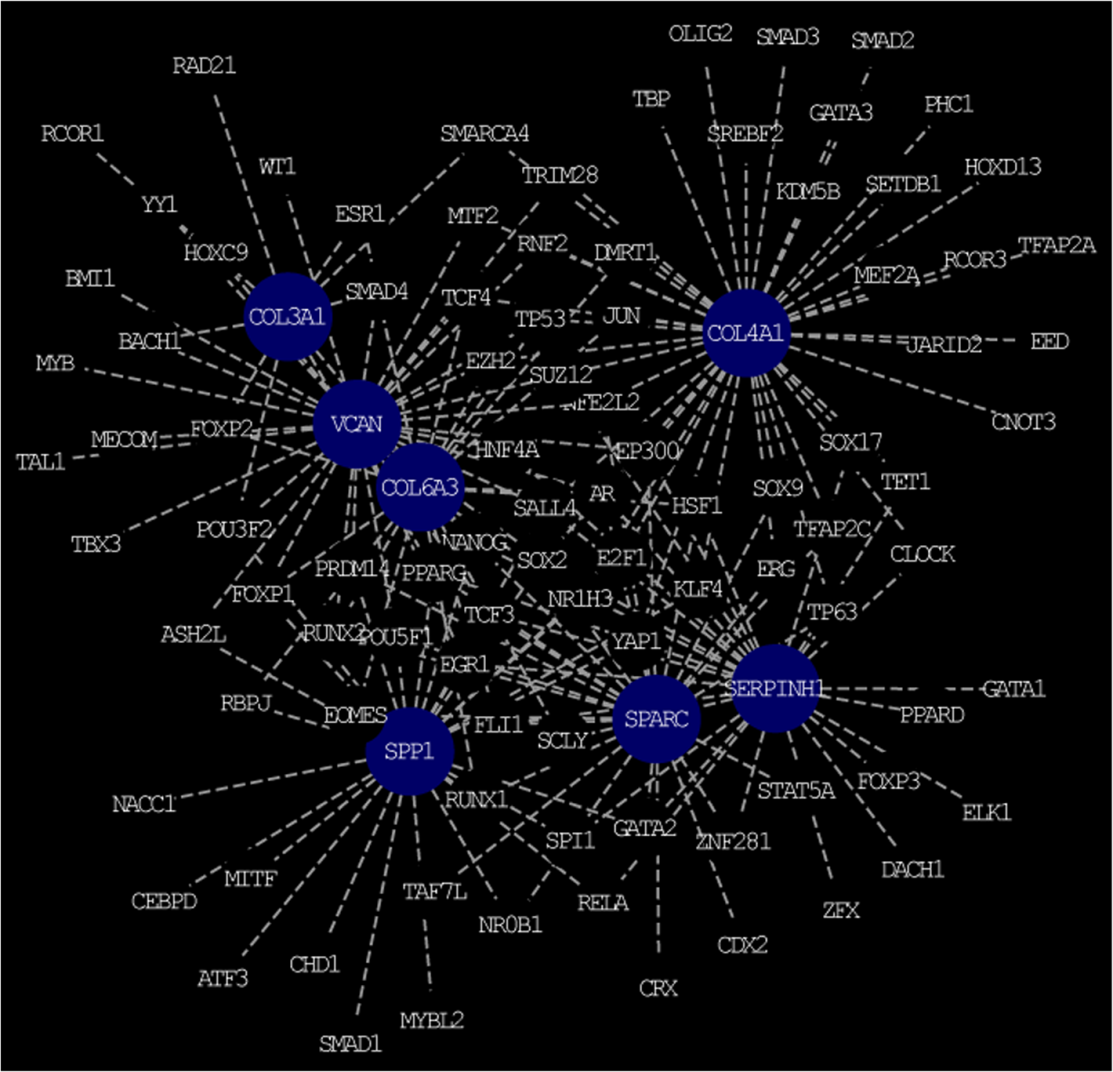
The network of transcription factors and hub genes

To further understand the regulatory network between TFs and hub genes, TFs with adj. *P* < 0.05 in ChEA through NetworkAnalyst were constructed by Cytoscape. As shown in Fig. 3, the transcription-regulated network with 182 edges and 102 nodes was obtained for hub genes. Different hub genes regulated by TFs are shown in Table 7, which androgen receptor (AR) had been predicted to regulate SPARC, COL6A3, SERPINH1, COL4A1, and VCAN, while COL4A1, VCAN, and COL6A3 could be regulated by EZH2.

**Table 7:** The transcription factors of hub genes

TFs	Genes	Total
AR	SPARC COL6A3 SERPINH1 COL4A1 VCAN	5
NANOG	SPARC SPP1 SERPINH1 COL3A1 VCAN	5
SOX2	SPARC SPP1 COL4A1 VCAN SERPINH1	5
EP300	SPARC COL4A1 VCAN SERPINH1	4
PPARG	SPARC SPP1 SERPINH1 COL3A1	4
SALL4	SPP1 COL4A1 VCAN SERPINH1	4
TCF3	SPARC SPP1 VCAN SERPINH1	4
EZH2	COL4A1 VCAN COL6A3	3
FLI1	SPARC SERPINH1 COL6A3	3
FOXP1	SPP1 VCAN COL6A3	3
GATA2	SPARC SPP1 SERPINH1	3
HSF1	SPARC SERPINH1 COL6A3	3
NFE2L2	SPARC COL4A1 VCAN	3
NR1H3	SPP1 COL4A1 COL6A3	3
POU3F2	SPP1 VCAN COL3A1	3
POU5F1	SPARC SPP1 VCAN	3
PRDM14	SPARC SPP1 VCAN	3
RUNX1	SPARC SPP1 COL6A3	3
RUNX2	SPARC VCAN COL6A3	3
SMAD4	VCAN COL6A3 COL3A1	3
SPI1	SPARC SPP1 SERPINH1	3
SUZ12	COL4A1 VCAN SERPINH1	3
TCF4	COL4A1 VCAN COL6A3	3
TP53	COL4A1 VCAN COL6A3	3
TP63	SPARC COL4A1 SERPINH1	3

### The prognostic value of hub genes

**Fig. 4:**
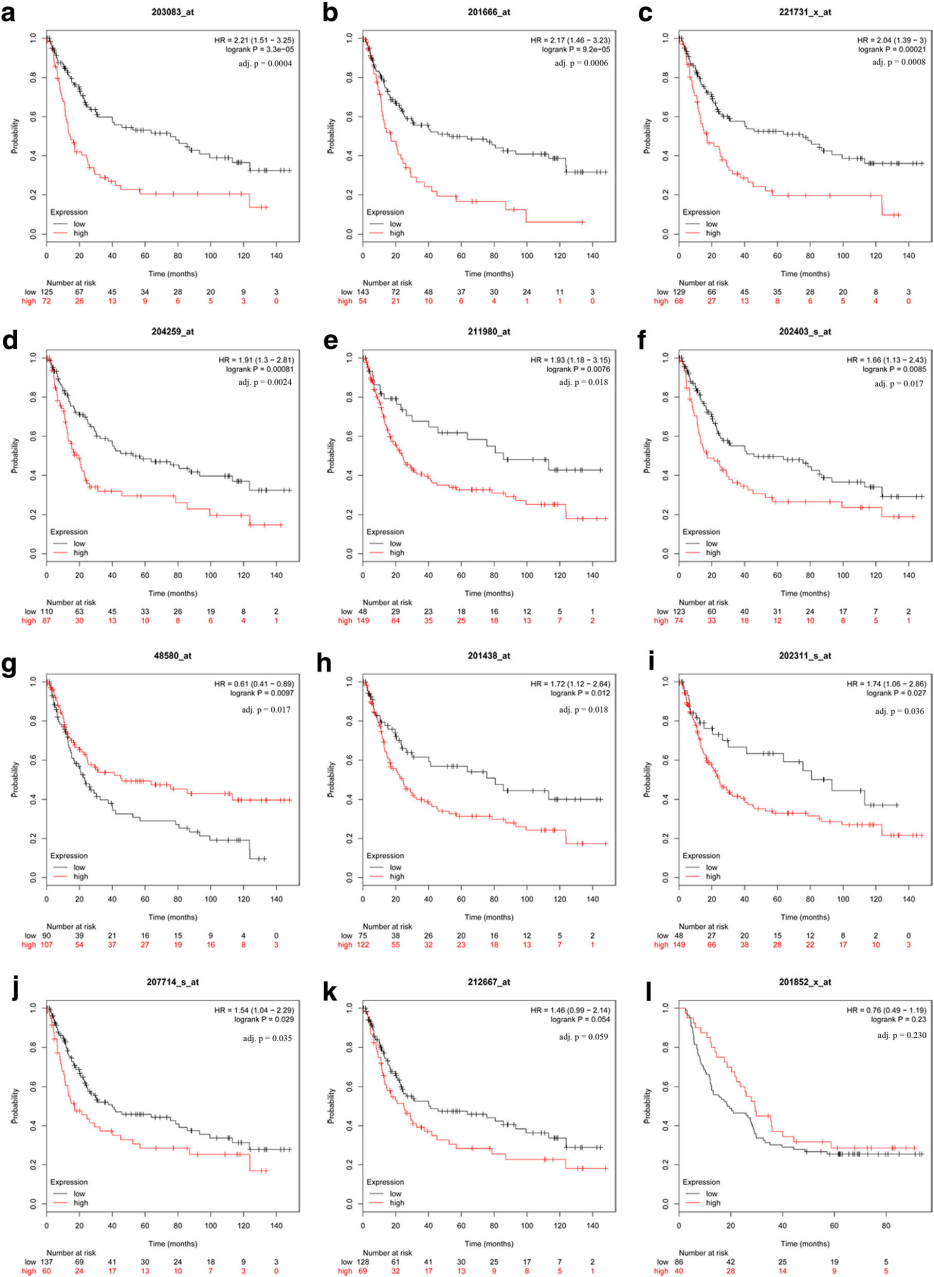
Prognostic value of 12 genes in GSE15459. Prognostic value in GSE15459 of THBS2(**a**), TIMP1(**b**), VCAN(**c**), MMP7(**d**), COL4A1(**e**), COL1A2(**f**), SPP1(**g**), COL6A3(**h**), COL1A1(**i**), SERINH1(**j**), SPARC(**k**), and COL3A1(**l**) were obtained in www.kmplot.com. The desired Affymetrix IDs are valid: 203083_at (THBS2), 201666_at (TIMP1), 221731_x_at (VCAN), 204259_at (MMP7), 211980_at (COL4A1), 202403_s_at (COL1A2), 48580_at (SPP1), 201438_at (COL6A3), 202311_s_at (COL1A1), 207714_s_at (SERPINH1), 212667_at (SPARC), and 201852_x_at (COL3A1). HR, hazard ratio; CI, confidence interval; adj. p, adjusted *p* value

**Fig. 5:**
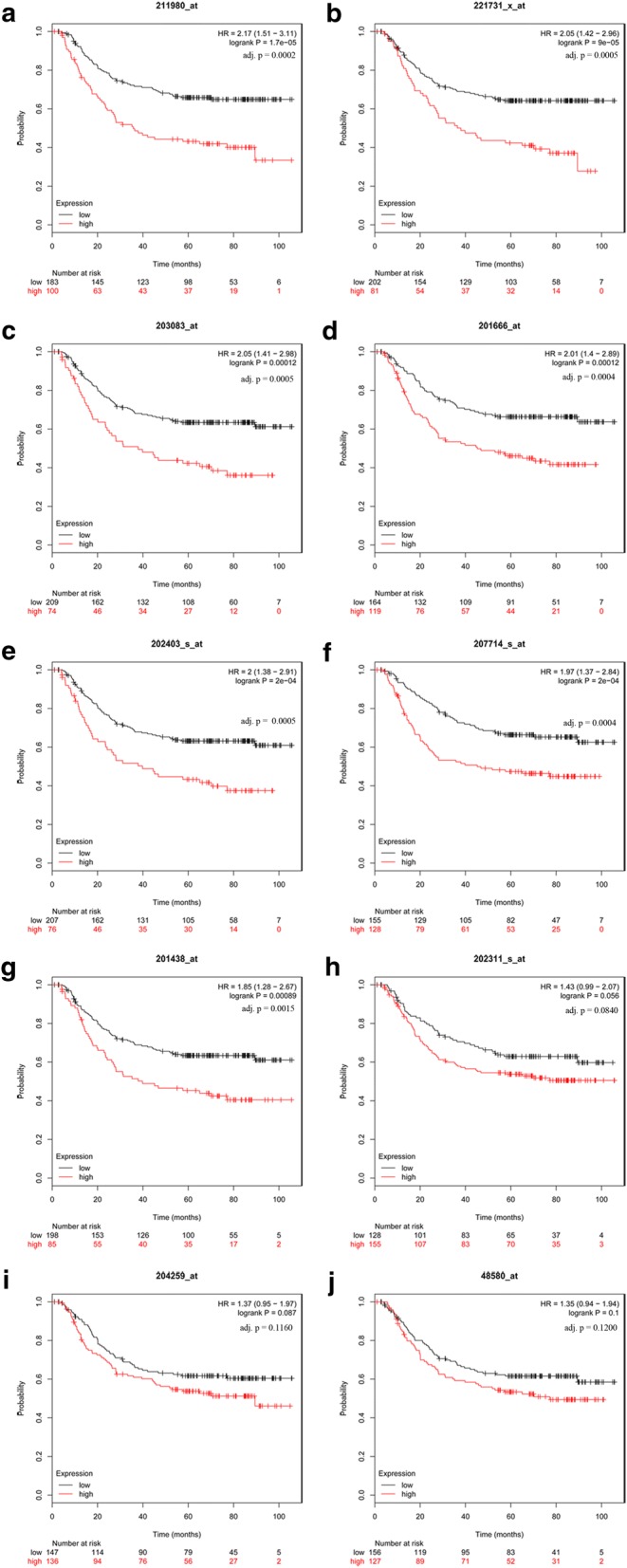
The validation of prognostic value of ten genes in GSE62254. Prognostic value in GSE62254 of COL4A1 (**a**), VCAN (**b**), THBS2 (**c**), TIMP1 (**d**), COL1A2 (**e**), SERINH1 (**f**), COL6A3 (**g**), COL1A1 (**h**), MMP7 (**i**), and SPP1 (**j**) were obtained in www.kmplot.com. The desired Affymetrix IDs are valid: 211980_at (COL4A1), 221731_x_at (VCAN), 203083_at (THBS2), 201666_at (TIMP1), 202403_s_at (COL1A2), 207714_s_at (SERPINH1), 201438_at (COL6A3), 202311_s_at (COL1A1), 204259_at (MMP7), and 48580_at (SPP1). HR, hazard ratio; CI, confidence interval; adj. p, adjusted *p* value

The prognostic value of 12 hub genes in PPI network was obtained from KM plotter (http://kmplot.com/analysis/). In consideration of the semblable histopathological feature and adequate quantity, the below two datasets were used to assess survival time. In GSE15459, it was found that high mRNA expression of SERPINH1 was associated with worse OS for GC patients [24, 25], as well as COL1A1, THBS2, COL4A1, COL6A3, MMP7, COL1A2, TIMP1, SPP1, and VCAN (Fig. 4). To validate the prognostic significance of these genes, the GSE62254 was analyzed [26]. In Fig. 5, seven out of ten genes had statistical significance, including COL4A1, VCAN, THBS2, TIMP1, COL1A2, SERPINH1, and COL6A3.

## Discussion

Due to the high heterogeneity of GC, GC was still a disease with high rates of prevalence and mortality. With surgery as the main, the other treatments including radiotherapy, chemotherapy, targeted therapy, and gene therapy as a supplement to the limited treatment measures of GC, the 5-year survival rate was still less than 30% [27]. Therefore, it is essential to explore the mechanisms of GC progression to prevent its occurrence, guide pharmacy, indicate the prognosis, or improve survival rate. The high-throughput platforms for detection of gene expression have been developing rapidly in diseases progression, which provides the basis of new targets discovery for diagnosis, therapy, and prognosis of cancers.

In this study, a total of 67 DEGs were screened, consisting of 36 upregulated genes and 31 downregulated genes. These upregulated genes were mainly enriched in angiogenesis and extracellular matrix organization, while downregulated genes were mainly involved in ion transport and response to bacterium in biological processes. Among these DEGs, 12 genes had high degrees in the PPI network. Following survival analysis of these genes revealed that 7 of these 12 upregulated genes were significantly correlated with worse overall survival of GC patients, including COL4A1, VCAN, THBS2, TIMP1, COL1A2, SERPINH1, and COL6A3.

The pathogenesis of cancer is multifactorial, with genetic, environmental, and lifestyle factors interacting to produce a given pathological characteristic. SERPINH1, also known as Hsp47, is a collagen-specific molecule that is essential for collagen synthesis [28]. The overexpression of SERPINH1 was found in many different cancers, including lung cancer, cervical squamous cancer, and glioma [29–31]. SERPINH1 may play an important role in tumor metastasis because of promoting maturation of various types of procollagens [32, 33]. Knockdown of SERPINH1 has been shown to significantly inhibit cell proliferation, migration, and invasion [31].

Type I collagen, including COL1A1 and COL1A2, is a major structural component of the ECM, and epithelial tumorigenesis is often accompanied by abnormal expression of ECM [34]. Overexpression of type I collagen was correlated with staging and poor disease-free survival of CRC patients [35]. It also found that type I collagen is required for maintaining lung cancer cell growth in 3D culture [36]. COL3A1 was a member of type III collagen and was found in extensible connective tissues. In epithelial ovarian cancers, the increase of COL3A1 was prognostic markers of poor prognosis [37]. High COL4A1 was revealed to be associated with advanced tumor stage as well as with bad overall and disease-free survival in HCC patients [38]. And COL4A1 knockdown led to cell viability reduction and cell cycle arrest in breast cancer cells [39]. COL6A3 has been observed to be frequently overexpressed in the GC tissues and also in five GC cell lines, including AGS, HGC-27, BGC-823, SGC-7901, and MGC-803 [40].

The other three hub genes are SPARC, SPP1, and VCAN. SPARC was overexpressed in highly metastatic tumors such as melanoma, breast cancer, and prostate cancer and acted as an anti-tumor factor in anti-angiogenesis, pro-apoptosis, cell proliferation inhibition, and cell cycle arrest in less metastatic tumors such as ovarian cancer, pancreatic cancer, colorectal cancer, and gastric cancer [41]. The high expression level of SPARC in GC tissues is controversial to its role in GC cells that it inhibited VEGF-induced proliferation and arrested cell cycle by reducing the activation of VEGFR2, ERK1/2, and AKT signaling pathways [42]. Therefore, SPARC may play different roles in different cancers and in different development stages of the same cancer. Elevated SPP1 levels have been detected in a variety of human cancers [43]; it may serve as a potential prognostic factor in GC [44]. The expression of SPP1 is related to the invasion and metastases of GC, and its mechanism may be to upregulate the expression of MMP-9 by activating NF-kappaB pathway [45]. VCAN was known to favor the homeostasis of the ECM [46]. And abolition of VCAN could reverse the increased migration effect which was induced by exogenous IL-11 in GC [47]. However, the oncogenic role and clinical significance of VCAN for GC were rarely explored. As the hub genes are mainly related to ECM, invasion and migration of GC seem to play a more important role in the development of cancer.

MiRNAs are known to regulate protein translation inhibition or targeted mRNA cleavage [48]. Increasing evidence suggested that miRNAs are involved in cancer development and progression, including gastric cancer [49]. In this study, we identified that hsa-miR-29c, hsa-miR-30c, hsa-miR-335, hsa-miR-33b, and hsa-miR-101 possibly affect the development and prognosis of gastric cancer through regulating the hub genes. MiR-29c expression was significantly decreased in GC [50]. MiR-29c downregulation was required to develop lung metastasis for the premetastatic CRC cells [51]. Recent study showed that hsa-miR-30c-5p was downregulated in GC tissues and remarkably related with lymphatic metastasis [52], and it suppressed the invasion ability of cancer by targeting metastasis-associated protein 1(MTA1) [52, 53]. MiR-30c was shown to be downregulated both in colon cancer specimens and prostate cancer [54, 55]. Aberrant expression of miR-335 is a noticeable factor in the cancer development [56, 57]. Furthermore, miR-335 suppressed the motibility of gastric cancer through regulating Bcl-w and specificity protein 1 [58]. MiR-33b was reported as a protective factor in multiple cancers [59–61]. In 2016, the methylation that resulted in downregulation of miR-33b was significantly detected in GC metastasis patients [62]. The genomic loss of miR-101 led to overexpression of EZH2, resulting in cancer progression [63, 64]. Therefore, this indicated that miRNAs play a noticeable role in the development and prognosis of GC through regulating disease-associated genes.

TF-hub genes regulatory network was constructed to explore the molecular mechanism of gastric cancer. In this study, we found AR, SOX2, EZH2, GATA2, RUNX1, SMAD4, and SUZ12 were meaningful for the expression of hub genes. It was reported that AR-negative patients had a significantly better survival than AR-positive GC patients. The SOX2 (sex-determining region Y-box 2) was a highly conserved transcription regulator and played a vital role in the cancer progression [65]. SOX2 was controversial in GC tissues; it was reported as a tumor promoter to activate AKT signaling on the one hand, and SOX2 was also detected as a protective factor to inhibit proliferation and metastasis [66, 67]. Overexpression of EZH2 was associated with poor prognosis and distant metastases in GC [68, 69]. As a potential metastasis-driving gene in prostate cancer [70], GATA2 was lack of research in GC. RUNX1 was reported as a target of AR, and its promoter was bound by EZH2 in prostate cancer [71]. Interestingly, RUNX1 played a cancer suppressor role in GC [72]. SMAD4 was frequently altered as common as TP53 in human gastric cancer [73]. SUZ12 is an essential component of Polycomb Repressive Complex2 (PRC2), affecting transcription by methylating histone and DNA [74]. Increased expression of SUZ12 was detected in GC and associated with pathological stage, metastasis, and poor prognosis [75]. The intricate interaction between TFs and hub genes made great contribution to the development of cancer.

## Conclusion

In summary, we intend to identify DEGs by bioinformatics analysis to find the potential biomarkers which may be involved in the progress of GC. The study provides a set of useful DEGs for future investigation into molecular mechanisms and biomarkers of GC. And the application of data mining and integration is available for prediction of GC progression. Nevertheless, further molecular biological explorations are required to verify the function of the DEGs in GC.

## Additional file

Additional file 1: Negatively correlated stem loop miRNAs of hub genes in a TCGA dataset composed of 380 gastric cancer tissues. (DOCX 30 kb)

## Abbreviations

BP: Biological process
DEGs: Differentially expressed genes
GC: Gastric cancer
OS: Overall survival
PPI: Protein-protein interaction
TFs: Transcription factors
