# Supplementary material for: Identification of hub genes with prognostic values in gastric cancer by bioinformatics analysis
Source: World J Surg Oncol. 2018 Jun 19;16:114. doi: 10.1186/s12957-018-1409-3 (PMC6009060; doi:10.1186/s12957-018-1409-3)
Supplement: Supplementary file 1 — Negatively correlated stem loop miRNAs of hub genes in a TCGA dataset composed of 380 gastric cancer tissues. (DOCX 30 kb) [file 12957_2018_1409_MOESM1_ESM.docx]

Additional file 1. Negatively correlated stem loop miRNAs of hub genes in a TCGA dataset composed of 380 gastric cancer tissues.

| Stem Loop ID | Gene | logFC | P Value | FDR | Stem Loop ID | Gene | logFC | P Value | FDR |
| --- | --- | --- | --- | --- | --- | --- | --- | --- | --- |
| hsa-let-7c | COL1A1 | -1.37102 | 5.53E-07 | 2.75E-06 | hsa-mir-196a-1 | COL1A2 | 5.173649 | 1.82E-87 | 8.23E-85 |
| hsa-mir-133a-2 | COL1A1 | -3.28029 | 8.77E-33 | 3.60E-31 | hsa-mir-196a-2 | COL1A2 | 3.993711 | 2.61E-37 | 1.47E-35 |
| hsa-mir-133b | COL1A1 | -3.43591 | 4.03E-41 | 3.64E-39 | hsa-mir-196b | COL1A2 | 4.826419 | 1.09E-77 | 2.45E-75 |
| hsa-mir-143 | COL1A1 | -1.8152 | 2.30E-07 | 1.30E-06 | hsa-mir-19a | COL1A2 | 1.169756 | 6.26E-08 | 4.16E-07 |
| hsa-mir-146b | COL1A1 | 1.571159 | 1.16E-12 | 1.94E-11 | hsa-mir-29c | COL1A2 | -1.45217 | 2.24E-08 | 1.63E-07 |
| hsa-mir-182 | COL1A1 | 1.483654 | 3.82E-07 | 2.03E-06 | hsa-mir-363 | COL1A2 | -1.6186 | 8.66E-12 | 1.22E-10 |
| hsa-mir-188 | COL1A1 | 1.584507 | 1.08E-10 | 1.28E-09 | hsa-mir-381 | COL1A2 | -1.04833 | 1.35E-07 | 7.92E-07 |
| hsa-mir-193a | COL1A1 | -1.06783 | 2.62E-07 | 1.46E-06 | hsa-mir-552 | COL1A2 | 3.718047 | 1.78E-27 | 6.72E-26 |
| hsa-mir-195 | COL1A1 | -1.38402 | 9.93E-12 | 1.36E-10 | hsa-mir-7-3 | COL1A2 | 1.551694 | 9.01E-08 | 5.74E-07 |
| hsa-mir-196a-1 | COL1A1 | 5.173649 | 1.82E-87 | 8.23E-85 | hsa-mir-767 | COL1A2 | 1.813376 | 1.47E-06 | 6.69E-06 |
| hsa-mir-196a-2 | COL1A1 | 3.993711 | 2.61E-37 | 1.47E-35 | hsa-let-7c | COL3A1 | -1.37102 | 5.53E-07 | 2.75E-06 |
| hsa-mir-196b | COL1A1 | 4.826419 | 1.09E-77 | 2.45E-75 | hsa-mir-196a-1 | COL3A1 | 5.173649 | 1.82E-87 | 8.23E-85 |
| hsa-mir-218-1 | COL1A1 | -1.13081 | 3.43E-04 | 1.00E-03 | hsa-mir-196a-2 | COL3A1 | 3.993711 | 2.61E-37 | 1.47E-35 |
| hsa-mir-218-2 | COL1A1 | -1.43116 | 2.98E-11 | 3.85E-10 | hsa-mir-196b | COL3A1 | 4.826419 | 1.09E-77 | 2.45E-75 |
| hsa-mir-28 | COL1A1 | -1.16295 | 6.34E-06 | 2.61E-05 | hsa-mir-29c | COL3A1 | -1.45217 | 2.24E-08 | 1.63E-07 |
| hsa-mir-29c | COL1A1 | -1.45217 | 2.24E-08 | 1.63E-07 | hsa-mir-33b | COL3A1 | 1.06251 | 3.36E-05 | 1.19E-04 |
| hsa-mir-328 | COL1A1 | -1.08873 | 4.85E-07 | 2.49E-06 | hsa-mir-381 | COL3A1 | -1.04833 | 1.35E-07 | 7.92E-07 |
| hsa-mir-335 | COL1A1 | 1.200402 | 3.43E-09 | 2.98E-08 | hsa-mir-767 | COL3A1 | 1.813376 | 1.47E-06 | 6.69E-06 |
| hsa-mir-503 | COL1A1 | 1.339988 | 1.17E-07 | 7.03E-07 | hsa-let-7c | COL4A1 | -1.37102 | 5.53E-07 | 2.75E-06 |
| hsa-mir-577 | COL1A1 | 1.286575 | 9.73E-07 | 4.63E-06 | hsa-mir-144 | COL4A1 | -1.6373 | 5.77E-14 | 1.09E-12 |
| hsa-mir-9-3 | COL1A1 | -2.10139 | 1.37E-10 | 1.55E-09 | hsa-mir-182 | COL4A1 | 1.483654 | 3.82E-07 | 2.03E-06 |
| hsa-mir-935 | COL1A1 | 1.490044 | 5.11E-07 | 2.59E-06 | hsa-mir-195 | COL4A1 | -1.38402 | 9.93E-12 | 1.36E-10 |
| hsa-mir-940 | COL1A1 | 1.252802 | 1.48E-06 | 6.69E-06 | hsa-mir-20b | COL4A1 | -1.4901 | 3.60E-10 | 3.96E-09 |
| hsa-mir-96 | COL1A1 | 1.771828 | 1.30E-12 | 2.10E-11 | hsa-mir-29c | COL4A1 | -1.45217 | 2.24E-08 | 1.63E-07 |
| hsa-let-7c | COL1A2 | -1.37102 | 5.53E-07 | 2.75E-06 | hsa-mir-381 | COL4A1 | -1.04833 | 1.35E-07 | 7.92E-07 |
| hsa-mir-105-2 | COL1A2 | 1.771443 | 1.22E-05 | 4.74E-05 | hsa-mir-503 | COL4A1 | 1.339988 | 1.17E-07 | 7.03E-07 |

Additional file 1. Negatively correlated stem loop miRNAs of hub genes in a TCGA dataset composed of 380 gastric cancer tissues.(continued)

| Stem Loop ID | Gene | logFC | P Value | FDR | Stem Loop ID | Gene | logFC | P Value | FDR |
| --- | --- | --- | --- | --- | --- | --- | --- | --- | --- |
| hsa-mir-767 | COL4A1 | 1.813376 | 1.47E-06 | 6.69E-06 | hsa-mir-200c | SERPINH1 | 1.123707 | 1.90E-04 | 5.73E-04 |
| hsa-mir-96 | COL4A1 | 1.771828 | 1.30E-12 | 2.10E-11 | hsa-mir-29c | SERPINH1 | -1.45217 | 2.24E-08 | 1.63E-07 |
| hsa-mir-1-1 | COL6A3 | -2.5258 | 9.04E-11 | 1.10E-09 | hsa-mir-429 | SERPINH1 | 1.550224 | 2.12E-09 | 1.95E-08 |
| hsa-mir-130b | COL6A3 | 1.334593 | 4.63E-09 | 3.88E-08 | hsa-mir-552 | SERPINH1 | 3.718047 | 1.78E-27 | 6.72E-26 |
| hsa-mir-139 | COL6A3 | -2.56445 | 2.25E-33 | 1.02E-31 | hsa-mir-940 | SERPINH1 | 1.252802 | 1.48E-06 | 6.69E-06 |
| hsa-mir-181b-2 | COL6A3 | 1.131267 | 1.90E-05 | 7.08E-05 | hsa-mir-101-2 | SPARC | -1.238 | 9.18E-10 | 8.83E-09 |
| hsa-mir-206 | COL6A3 | -1.79548 | 1.21E-07 | 7.18E-07 | hsa-mir-105-2 | SPARC | 1.771443 | 1.22E-05 | 4.74E-05 |
| hsa-mir-28 | COL6A3 | -1.16295 | 6.34E-06 | 2.61E-05 | hsa-mir-125a | SPARC | -1.12163 | 3.26E-07 | 1.77E-06 |
| hsa-mir-29c | COL6A3 | -1.45217 | 2.24E-08 | 1.63E-07 | hsa-mir-125b-2 | SPARC | -1.19928 | 5.37E-07 | 2.70E-06 |
| hsa-mir-301b | COL6A3 | 1.517523 | 8.41E-07 | 4.04E-06 | hsa-mir-146b | SPARC | 1.571159 | 1.16E-12 | 1.94E-11 |
| hsa-mir-335 | COL6A3 | 1.200402 | 3.43E-09 | 2.98E-08 | hsa-mir-18a | SPARC | 1.797128 | 1.18E-15 | 2.67E-14 |
| hsa-mir-486 | COL6A3 | -1.98119 | 7.03E-19 | 2.12E-17 | hsa-mir-192 | SPARC | 1.719582 | 2.27E-06 | 1.01E-05 |
| hsa-mir-579 | COL6A3 | 1.003594 | 1.53E-03 | 3.86E-03 | hsa-mir-193a | SPARC | -1.06783 | 2.62E-07 | 1.46E-06 |
| hsa-mir-605 | COL6A3 | -1.70801 | 7.04E-07 | 3.42E-06 | hsa-mir-19a | SPARC | 1.169756 | 6.26E-08 | 4.16E-07 |
| hsa-mir-767 | COL6A3 | 1.813376 | 1.47E-06 | 6.69E-06 | hsa-mir-204 | SPARC | -2.51743 | 6.66E-24 | 2.32E-22 |
| hsa-mir-877 | COL6A3 | 1.490746 | 3.27E-08 | 2.31E-07 | hsa-mir-20b | SPARC | -1.4901 | 3.60E-10 | 3.96E-09 |
| hsa-mir-181b-2 | MMP7 | 1.131267 | 1.90E-05 | 7.08E-05 | hsa-mir-215 | SPARC | 1.685801 | 1.34E-06 | 6.24E-06 |
| hsa-mir-194-2 | MMP7 | 1.858527 | 1.20E-08 | 9.33E-08 | hsa-mir-222 | SPARC | 1.229276 | 3.90E-09 | 3.32E-08 |
| hsa-mir-19a | MMP7 | 1.169756 | 6.26E-08 | 4.16E-07 | hsa-mir-28 | SPARC | -1.16295 | 6.34E-06 | 2.61E-05 |
| hsa-mir-381 | MMP7 | -1.04833 | 1.35E-07 | 7.92E-07 | hsa-mir-29c | SPARC | -1.45217 | 2.24E-08 | 1.63E-07 |
| hsa-mir-125a | SERPINH1 | -1.12163 | 3.26E-07 | 1.77E-06 | hsa-mir-378 | SPARC | -1.3524 | 5.96E-09 | 4.90E-08 |
| hsa-mir-1304 | SERPINH1 | 1.158205 | 1.16E-04 | 3.78E-04 | hsa-mir-504 | SPARC | -1.34318 | 4.57E-06 | 1.93E-05 |
| hsa-mir-133a-2 | SERPINH1 | -3.28029 | 8.77E-33 | 3.60E-31 | hsa-mir-509-3 | SPARC | 1.375675 | 4.06E-04 | 1.15E-03 |
| hsa-mir-133b | SERPINH1 | -3.43591 | 4.03E-41 | 3.64E-39 | hsa-mir-549 | SPARC | 2.68146 | 2.46E-09 | 2.22E-08 |
| hsa-mir-188 | SERPINH1 | 1.584507 | 1.08E-10 | 1.28E-09 | hsa-mir-552 | SPARC | 3.718047 | 1.78E-27 | 6.72E-26 |
| hsa-mir-200b | SERPINH1 | 1.515505 | 8.67E-08 | 5.60E-07 | hsa-mir-556 | SPARC | 1.284855 | 3.83E-05 | 1.34E-04 |

Additional file 1. Negatively correlated stem loop miRNAs of hub genes in a TCGA dataset composed of 380 gastric cancer tissues.(continued)

| Stem Loop ID | Gene | logFC | P Value | FDR | Stem Loop ID | Gene | logFC | P Value | FDR |
| --- | --- | --- | --- | --- | --- | --- | --- | --- | --- |
| hsa-mir-579 | SPARC | 1.003594 | 1.53E-03 | 3.86E-03 | hsa-mir-549 | THBS2 | 2.68146 | 2.46E-09 | 2.22E-08 |
| hsa-mir-592 | SPARC | 2.046905 | 8.07E-14 | 1.46E-12 | hsa-mir-579 | THBS2 | 1.003594 | 1.53E-03 | 3.86E-03 |
| hsa-mir-767 | SPARC | 1.813376 | 1.47E-06 | 6.69E-06 | hsa-mir-592 | THBS2 | 2.046905 | 8.07E-14 | 1.46E-12 |
| hsa-mir-935 | SPARC | 1.490044 | 5.11E-07 | 2.59E-06 | hsa-mir-767 | THBS2 | 1.813376 | 1.47E-06 | 6.69E-06 |
| hsa-mir-181b-2 | SPP1 | 1.131267 | 1.90E-05 | 7.08E-05 | hsa-mir-802 | THBS2 | -1.30525 | 2.98E-03 | 6.91E-03 |
| hsa-mir-20b | SPP1 | -1.4901 | 3.60E-10 | 3.96E-09 | hsa-mir-885 | THBS2 | -1.99205 | 5.00E-08 | 3.37E-07 |
| hsa-mir-577 | SPP1 | 1.286575 | 9.73E-07 | 4.63E-06 | hsa-mir-9-3 | THBS2 | -2.10139 | 1.37E-10 | 1.55E-09 |
| hsa-mir-579 | SPP1 | 1.003594 | 1.53E-03 | 3.86E-03 | hsa-mir-935 | THBS2 | 1.490044 | 5.11E-07 | 2.59E-06 |
| hsa-mir-135b | THBS2 | 3.186272 | 3.21E-39 | 2.07E-37 | hsa-mir-940 | THBS2 | 1.252802 | 1.48E-06 | 6.69E-06 |
| hsa-mir-142 | THBS2 | 1.26474 | 2.92E-06 | 1.25E-05 | hsa-mir-96 | THBS2 | 1.771828 | 1.30E-12 | 2.10E-11 |
| hsa-mir-144 | THBS2 | -1.6373 | 5.77E-14 | 1.09E-12 | hsa-mir-101-2 | VCAN | -1.238 | 9.18E-10 | 8.83E-09 |
| hsa-mir-182 | THBS2 | 1.483654 | 3.82E-07 | 2.03E-06 | hsa-mir-129-2 | VCAN | -2.0211 | 8.09E-16 | 1.92E-14 |
| hsa-mir-188 | THBS2 | 1.584507 | 1.08E-10 | 1.28E-09 | hsa-mir-135b | VCAN | 3.186272 | 3.21E-39 | 2.07E-37 |
| hsa-mir-195 | THBS2 | -1.38402 | 9.93E-12 | 1.36E-10 | hsa-mir-144 | VCAN | -1.6373 | 5.77E-14 | 1.09E-12 |
| hsa-mir-204 | THBS2 | -2.51743 | 6.66E-24 | 2.32E-22 | hsa-mir-192 | VCAN | 1.719582 | 2.27E-06 | 1.01E-05 |
| hsa-mir-20b | THBS2 | -1.4901 | 3.60E-10 | 3.96E-09 | hsa-mir-200a | VCAN | 1.69807 | 1.84E-09 | 1.74E-08 |
| hsa-mir-21 | THBS2 | 1.524249 | 1.08E-07 | 6.78E-07 | hsa-mir-215 | VCAN | 1.685801 | 1.34E-06 | 6.24E-06 |
| hsa-mir-222 | THBS2 | 1.229276 | 3.90E-09 | 3.32E-08 | hsa-mir-579 | VCAN | 1.003594 | 1.53E-03 | 3.86E-03 |
| hsa-mir-29c | THBS2 | -1.45217 | 2.24E-08 | 1.63E-07 | hsa-mir-767 | VCAN | 1.813376 | 1.47E-06 | 6.69E-06 |
| hsa-mir-30a | THBS2 | -1.65228 | 2.91E-09 | 2.58E-08 |  |  |  |  |  |
| hsa-mir-30c-2 | THBS2 | -1.01405 | 2.80E-06 | 1.22E-05 |  |  |  |  |  |
| hsa-mir-497 | THBS2 | -1.05245 | 3.63E-07 | 1.96E-06 |  |  |  |  |  |
| hsa-mir-509-2 | THBS2 | 1.484827 | 2.13E-04 | 6.26E-04 |  |  |  |  |  |
| hsa-mir-509-3 | THBS2 | 1.375675 | 4.06E-04 | 1.15E-03 |  |  |  |  |  |
